# X-ray diffraction strain analysis of a single axial InAs_1–*x*_P_*x*_ nanowire segment

**DOI:** 10.1107/S160057751402284X

**Published:** 2015-01-01

**Authors:** Mario Keplinger, Bernhard Mandl, Dominik Kriegner, Václav Holý, Lars Samuelsson, Günther Bauer, Knut Deppert, Julian Stangl

**Affiliations:** a Solid State and Semiconductor Physics, Johannes Kepler University Linz, A-4040 Linz, Austria; b Solid State Physics, Lund University, S-22 100 Lund, Sweden; c Faculty of Mathematics and Physics, Charles University, Ke Karlovu 5, 121 16 Prague, Czech Republic

**Keywords:** nanowire, nano-focus, strain analysis, hetero-structure, finite-element simulation

## Abstract

The spatial strain distribution in and around a single axial InAs_1–*x*_P_*x*_ hetero-segment in an InAs nanowire was analyzed using nano-focused X-ray diffraction. In connection with finite-element-method simulations a detailed quantitative picture of the nanowire’s inhomogeneous strain state was achieved. This allows for a detailed understanding of how the variation of the nanowire’s and hetero-segment’s dimensions affect the strain in its core region and in the region close to the nanowire’s side facets. Moreover, ensemble-averaging high-resolution diffraction experiments were used to determine statistical information on the distribution of wurtzite and zinc-blende crystal polytypes in the nanowires.

## Abstract 2

Strain analysis of an axial InAs_1–*x*_P_*x*_ hetero-segment in an InAs nanowire using nano-focused X-ray diffraction is presented.

## 1. Introduction   

For the fabrication of future electronic and opto-electronic devices, investigations of new device designs made from precisely tailored semiconductor materials are crucial. Nanowires (NWs), for instance, offer a great flexibility of altering their material’s electronic and optoelectronic properties in beneficial ways. Being one-dimensional in the quantum mechanical sense renders their physical properties different from three-dimensional bulk. Moreover, by including axial hetero-segments of different materials, the formation of zero-dimensional quantum dots or tunnel barriers becomes possible. Elastic relaxation due to the small diameter allows for larger lattice mismatches between materials, as compared with two-dimensional layer systems, during fabrication without the introduction of defects. This further enhances the design freedom as has been shown in previous works (Ganjipour *et al.*, 2012 ▶; Borgström *et al.*, 2011 ▶; Wallentin *et al.*, 2010 ▶; Roddaro *et al.*, 2011 ▶; Reimer *et al.*, 2012 ▶; Romeo *et al.*, 2012 ▶). Inhomogeneous strain fields are intimately connected to such heterostructures, which can alter the band structure of semiconductor materials dramatically. This can be exploited, for example, to enhance electron mobility (Chu *et al.*, 2009 ▶; Hrauda *et al.*, 2011 ▶) or to alter electro-optical properties (Greil *et al.*, 2012 ▶). Control on the details of strain distributions in nanostructures is thus an important task in device fabrication, which in turn requires precise analysis tools to monitor those strain fields. In this work we present a detailed strain characterization of InAs NWs containing a short axial InAs_1–*x*_P_*x*_ segment. Both ensemble-average experiments as well as the analysis of a single NW using nano-focused synchrotron radiation have been carried out. The NWs are not only strained due to the lattice mismatch of the hetero-segment; strain is also introduced from a stacking of wurtzite (WZ) and zinc-blende (ZB) polytypes, since the wurtzite polytype has a slightly different lattice spacing than zinc-blende (Kriegner *et al.*, 2011*a* ▶,*b* ▶). High-resolution X-ray diffraction (HRXRD) measurements on ensembles of the as-grown heterostructured NWs were used to obtain the average WZ/ZB polytype segment length and the wurtzite content. With nano-focused X-ray diffraction (XRD) we precisely monitored the chemical composition and dimension of the investigated InAs_1–*x*_P_*x*_ segment. The NW’s chemical composition and dimension define the strain distribution in the region inside and around the hetero-segment. Nano-focused XRD allows for illuminating a single InAs_1–*x*_P_*x*_ segment of one NW and to record the diffracted intensity of the segment and its vicinity, from which its three-dimensional strain distribution is found. The WZ/ZB polytypism needs to be considered in the analysis to yield correct results. With the presented method a precise knowledge of the spatial strain variation is gained, a prerequisite for the fabrication of novel strain engineered NW devices.

## 2. Sample fabrication   

**Figure 1:**
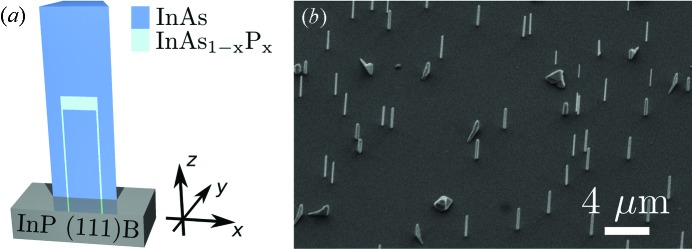
(*a*) Sketch of the fabricated NW with embedded InAs_1–*x*_P_*x*_ hetero-segment; the two different colours denote the two different material combinations. The coordinate system used in this work is denoted with respect to the NW in the bottom right of the sketch. (*b*) Scanning electron microscope image recorded at a sample tilt of 45°.

The NW fabrication followed in principle the self-seeded In particle-assisted growth scheme reported by Mandl *et al.* (2006 ▶) ▶ and Mandl *et al.* (2010 ▶) ▶. InP (111)B substrates were covered with a ∼13 Å thin SiO_*x*_ layer (*x* ≃ 1). This deposition was carried out using thermal sublimation and the layer thickness was controlled by an oscillating quartz system mounted in the deposition chamber. During all growth steps the temperature was fixed to 853 K, and H_2_ was used as carrier gas with a flow rate of 13000 ml min^−1^. By using a special switching sequence of gas precursors in an Aixtron 200/4 metal-organic vapour phase epitaxy reactor, an InAs_1–*x*_P_*x*_ segment was embedded in an InAs NW. First the InAs core NW was grown for 2 min with a trimethylindium and arsine precursor molar fraction of 1.1 × 10^−5^ and 3.8 × 10^−4^. Then InAs_1–*x*_P_*x*_ growth was initiated for 10 s with a phosphine and arsine precursor molar fraction of 6.2 × 10^−3^ and 1.9 × 10^−4^. Finally, a second InAs segment was grown under the same conditions as the first one. Fig. 1(*a*) ▶ shows an illustration of the fabricated structure, where additional radial shells, due to a finite radial growth rate typical of the self-seeded particle-assisted mechanism, are indicated. However, these shells are extremely thin compared with the axial segment lengths. From a detailed growth study (to be published elsewhere) we found a rather wide distribution of shell thicknesses within the NW ensemble from only a few nanometres up to 20 nm.

The coordinate system used later on is also depicted in the inset of Fig. 1(*a*) ▶: the NW growth direction, the cubic [111] direction, is depicted as the *z*-direction. The in-plane directions *x* and *y* in the substrate surface are along the crystallographic [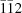] and [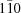] directions, respectively.

## 3. Ensemble characterization experiments   

For a first characterization of the NW shape and size, scanning electron microscope (SEM) images were recorded in a Jeol 6400F system, shown in Fig. 1(*b*) ▶. An average NW diameter of 180 ± 40 nm and an average NW length of 3.8 ± 0.7 µm were found. This means that the NWs on the sample exhibit a certain variation of their geometric properties within the as-grown ensemble. The hetero-segment is not visible in the SEM micrographs, and also the WZ/ZB stacking properties cannot be obtained.

**Figure 2:**
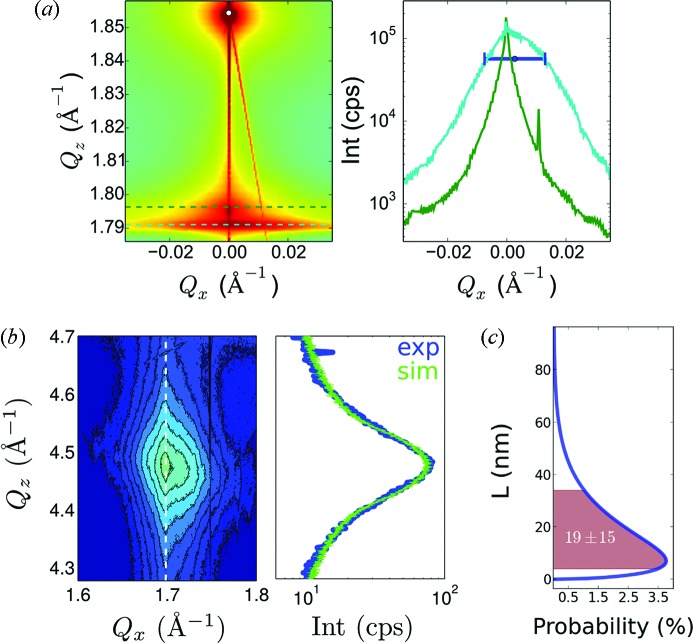
(*a*) Reciprocal-space map of the symmetric HRXRD measurement around the (111) InAs/InAs_1–*x*_P_*x*_ Bragg reflections. The InP Bragg reflection is denoted with a white circle. Furthermore, two slices are shown in the graph on the right-hand side at the *Q*_*z*_ positions of the dashed lines indicated in the reciprocal-space map. The green line is positioned at the nominal position of the (111) InAs zinc-blende Bragg reflection, and the cyan line at the (111) and its hexagonal equivalent (000.2) InAs WZ/ZB Bragg reflections, with 70% WZ and 30% ZB. The position as well as the FWHM of a Gauss distribution fitted to the WZ/ZB Bragg reflection are shown as a blue error bar in the right-hand graph. (*b*) Reciprocal-space map of the asymmetric HRXRD measurement. The contour plot shows the intensity distribution around the (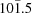) InAs/InAs_1–*x*_P_*x*_ Bragg reflections, and the dashed white line indicates the position of the cut shown beside; the blue line represents the measured intensity, the green line the simulation. (*c*) The Gamma distribution function which describes the distribution of the WZ segment lengths (*L*) in the investigated NWs determined by the simulation shown in (*b*).

For the NW ensemble’s X-ray characterization, intensity distributions around the symmetric (111) and asymmetric (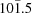) InAs/InAs_1–*x*_P_*x*_ Bragg reflections^1^ were recorded at the HRXRD beamline BW2 at HASYLAB in Hamburg, Germany, using an X-ray energy of 9.5 keV. The recorded reciprocal-space map around the (111) InAs/InAs_1–*x*_P_*x*_ signal is shown in Fig. 2(*a*), and Fig. 2(*b*) ▶ shows the intensity distribution around the hexagonal (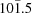) peak. Note that no information on the InAs_1–*x*_P_*x*_ segment and the thin shells is gained from the ensemble HRXRD experiments, since their scattering volume is too small compared with the entire NW volume to contribute significantly to the diffraction signal. However, the fraction of WZ material in the NWs and the average WZ segment length can be deduced from this experiment.

The (111) Bragg reflection is allowed for both ZB and WZ structure, for which it corresponds to the (000.2) hexagonal Bragg reflection. Therefore, it is sensitive to the *d*-spacing distribution in the [111] direction of the whole NWs, but almost insensitive to the WZ/ZB stacking sequence. The only influence of the stacking is indirect *via* the slightly different lattice parameters for ZB and WZ. This leads to shifts of the NW’s Bragg peak position as a function of the overall WZ/ZB ratio and a significantly smaller broadening in the *Q*_*z*_ direction as for the (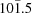) reflection discussed below. The position of the (111) NW Bragg reflection is therefore a way to determine the *total fraction* of WZ segments in the NW. The average NW ensemble lattice parameter and its variation due to the WZ/ZB polytypes was calculated using finite-element-method (FEM) simulations and was then compared with the (111) NW Bragg reflection’s position and shape in the *Q*_*z*_ direction, which resulted in a WZ content of around 70%. Detailed explanation of the FEM simulations, which have been performed using a commercial software package, will be given in the next section. The *Q*_*z*_ positions of pure zinc-blende InAs and WZ/ZB mixture are indicated in Fig. 2(*a*) ▶. Moreover, a cut through the NW signal is plotted which shows an asymmetry of the (111) NW Bragg reflection in the *Q*_*x*_ direction. This is also seen in the intensity profile of the (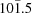) Bragg reflection, see Fig. 2(*b*) ▶, which stems from an asymmetric tilt distribution of the NWs on the sample. Such NW tilting and bending have been discussed by Keplinger *et al.* (2010 ▶) ▶, and possible reasons are defects at the substrate interface or an inhomogeneous thickness of the grown shell in heterostructured NWs. From the evaluation of the (111) Bragg peak position as well as its FWHM in the [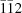] direction we calculated a tilt distribution of ±0.3° around an offset tilt angle of ±0.1°.

For the (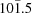) Bragg reflection shown in Fig. 2(*b*) ▶, the parts of the sample having WZ crystal structure provide the major amount of scattered intensity, except for a small, almost constant, contribution from the crystal truncation rod of the cubic (224) reflection, which is already far away at *Q*_*z*_ = 4.8 Å^−1^. Since we are not dependent on the absolute intensity values in the data evaluation but only the peak shape, this crystal truncation rod contribution to the intensity of the (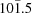) Bragg reflection can be neglected. From a reference measurement (to be published elsewhere) we confirmed that the WZ phase occurs only within NWs on this sample, and not from a possible two-dimensional layer, as well as rather bulky crystallites on the sample visible in Fig. 1(*b*) ▶, which are purely ZB. This finding confirms earlier studies, *e.g.* by Eymery *et al.* (2007 ▶) ▶ and Kriegner *et al.* (2011*a* ▶) ▶, who also find WZ phase exclusively in NWs. In the former case, truncation rod analysis in grazing-incidence geometry is used to disentangle planar overgrowth in NW material.

For a single NW with alternating WZ/ZB segments the stacking gives rise to a special speckle pattern of hexagonal Bragg peaks (Favre-Nicolin *et al.*, 2009 ▶; Chamard *et al.*, 2009 ▶), which is nothing else than the Fourier transformation of the particular WZ distribution function along this particular NW. The average length of the WZ segments defines the speckle pattern’s envelope function’s full width at half-maximum (FWHM) in the *Q*_*z*_ direction. When performing ensemble measurements as in this case, *i.e.* illuminating a large number of NWs, the recorded intensity distribution is an incoherent superposition of intensities originating from various NWs. This ‘smears out’ the speckle pattern and eventually its envelope defines the shape of the Bragg reflection, as observed in Fig. 2(*b*) ▶.

To simulate the shape function of the (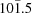) Bragg reflection in the *Q*_*z*_ direction, a Monte Carlo approach was used: arbitrary stacks of WZ and ZB segments with randomly varied lengths were assembled using computer-generated pseudo random numbers. This results in WZ and ZB segment lengths distributed according to a Gamma function with a certain mean length and variation. Then, the ZB and WZ segments were assigned with a scattering density according to 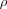 = 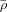 ± (Δρ_WZ/ZB_/2). The mean scattering density 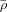 was set to zero to avoid very sharp and intense features at zero frequency in Fourier space, since the simulations should just describe the shape of the scattered intensity distribution. From such WZ/ZB distribution functions simulated diffraction patterns of the hexagonal (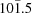) Bragg reflection were then calculated using a discrete Fourier transformation. By calculating the arithmetic average of many statistically varied simulated diffraction patterns the real situation of many incoherently scattering wires within the illuminated volume was simulated. To verify the statistical quality of this Monte Carlo approach, *i.e.* ensuring that the resulting signal’s properties only depend on the average length as well as the length variation of the simulated WZ segments, the relative root-mean-square deviation 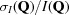 was calculated for each Monte Carlo step:
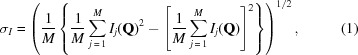
where 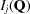 is the *j*th simulated intensity distribution and *M* is the total number of simulations. With *M* = 150 simulated individual ZB/WZ stacks a max[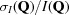] far below 1% was obtained. Quantitative results were then found by varying the Gamma distribution function parameters, *i.e.* the average WZ segment length and its variation, until a good correspondence with the measured intensity pattern was obtained. Fig. 2(*b*) ▶ shows the measured two-dimensional intensity distribution of the (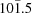) InAs/InAs_1–*x*_P_*x*_ Bragg reflection along with the simulated shape function (green) compared with the measured intensity distribution (blue) at the position of the white dashed line. The resulting wurtzite segment length distribution parameters are: a mean length of 19 nm with a σ of 15 nm, see Fig. 2(*c*) ▶.

## 4. Investigation of a single InAs_1–*x*_P_*x*_ segment   

**Figure 3:**
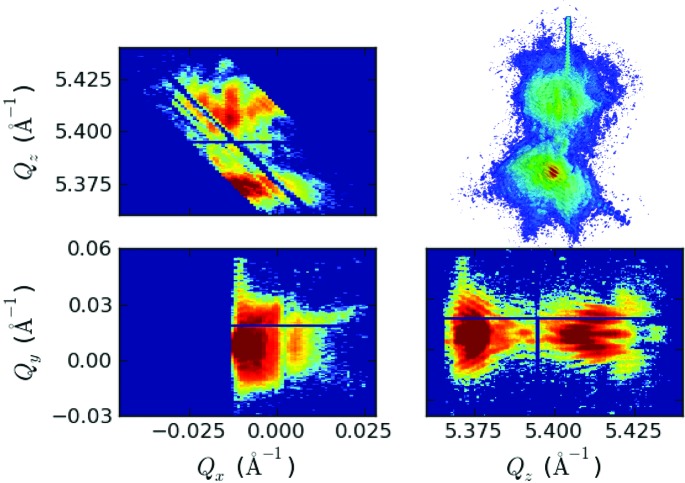
Three-dimensional reciprocal-space map from the nano-focus XRD experiment. This graph shows the three-dimensional intensity distribution around the (333) Bragg reflection of the InAs_1–*x*_P_*x*_ region inside a single NW and slices through the point of maximum intensity along all three axes.

To gain further insight into the investigated structures and in particular into the short InAs_1–*x*_P_*x*_ hetero-segment, we performed nano-focused X-ray diffraction experiments at beamline ID01 at the ESRF in Grenoble, France, at an X-ray energy of 10.2 keV. By using a Fresnel zone plate the X-ray beam was focused down to a FWHM of 200 nm × 350 nm. With such a nano-focused beam, only a small part of a single NW is illuminated. To align the InAs_1–*x*_P_*x*_ hetero-segment of a single wire into the focal spot of the X-ray beam, first a rough pre-alignment of the sample with an optical microscope, mounted on the goniometer, was performed. However, in this way the position of the X-ray spot on the sample can only be determined within a precision of a few micrometres. A finer alignment followed using scanning diffraction microscopy, as described by Diaz *et al.* (2009 ▶) ▶: by setting the detector and sample angle being sensitive to the NW signal, one records the intensity while moving the sample laterally. This gives a real-space map of the NW positions with a resolution according to the focused X-ray beam size. After finding and selecting one NW with this method and moving the X-ray spot along the NW to find the hetero-segment position, the scattered intensity was recorded using a two-dimensional MAXIPIX detector by rocking the sample around the (333) InAs/InAs_1–*x*_P_*x*_ Bragg reflection. This results in the measurement of a three-dimensional reciprocal-space map (RSM). After every sample rocking step of 0.01° a realignment of the InAs_1–*x*_P_*x*_ segment into the centre of the beam, due to a minimal sample drift with respect to the X-ray beam, was necessary, which was carried out using two lateral sample movement scans. Fig. 3 ▶ shows the measured intensity distribution as a three-dimensional contour plot and additionally as two-dimensional contour plots showing slices through the point of maximum intensity along all three principle axes. In the 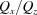 and 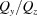 slices as well as in the three-dimensional illustration of the data the intensity maximum around 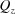 = 5.375 Å^−1^ is attributed to the InAs part of the illuminated real-space volume, whereas the broader signal around 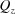 = 5.41 Å^−1^ comes from the InAs_1–*x*_P_*x*_ segment. Moreover, in the 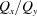 slice facet streaks with thickness oscillations from the NW side facets are observed. The InP (333) substrate peak, which would occur at 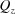 = 5.563 Å^−1^, is outside the plotted *Q* range. The (333) Bragg reflection is sensitive to the *d*-spacing distribution only along the [111] direction. However, due to the known symmetric shape of the NWs, the other strain components become linked to the strain along the [111] direction, so that performing finite-element calculations and simulations of the resulting intensity distribution around the (333) reflection actually allow conclusions to be drawn also on the strain components in the *x* and *y* directions. To deduce the spatial strain distribution, FEM calculations and kinematical scattering simulations were used to reproduce the measured RSMs. The NW was simulated as a three-dimensional object with the outer dimensions taken from SEM measurements. The phosphorous content in the InAs_1–*x*_P_*x*_ segment was then introduced by applying initial strain according to the lattice mismatch of InAs and InAs_1–*x*_P_*x*_ in the heterostructure part of the simulated NW. Additionally, arbitrarily distributed wurtzite and zinc-blende segments were mimicked by randomly distributing wurtzite segments, with the same dimensions and distribution function as obtained from the simulations of the ensemble measurements. As an initial strain state of these wurtzite segments, anisotropic strain according to the lattice parameter deviations reported by Kriegner *et al.* (2011*a* ▶) ▶ for WZ InAs and by Kriegner *et al.* (2011*b* ▶) ▶ for WZ InP was used. The lattice parameters as well as the elastic constants of cubic InAs and InP were taken from IOFFE (2014 ▶). Elastic constants were transformed according to Hirth & Lothe (1992 ▶) and Martin (1972 ▶) for ZB and WZ polytypes, respectively. For the lattice parameter variation of InAs_1–*x*_P_*x*_ according to the P content, Vegard’s law was used. With the simulated displacements field from FEM^2^ the diffusely scattered intensity was calculated using kinematical scattering theory.

## 5. Discussion   

The parameter space of the simulations was spanned by the InAs_1–*x*_P_*x*_ segment thickness, its phosphorous concentration, the thickness of the InAs and InAs_1–*x*_P_*x*_ shells, the outer diameter of the NW, and finally the WZ/ZB segment structure. Also the illumination of only a part of the NW by the focused beam needs to be taken into account. First, to narrow down the parameter space, a range of reasonable parameter values was mapped in a series of simulations, where the agreement between measurement and simulation was judged ‘guided by the eye’. After the relevant range of parameter space was found, the accuracy of the simulations was additionally judged by calculating the sum of squared residuals for every simulation. The whole procedure has been iterated several times to find the optimum values for all parameters.

The outer wire diameter was initially chosen according to SEM micrographs and slightly varied around this value. The simulated thickness fringes depend very sensitively on the NW diameter, so that it could be fixed to 175 nm. An initial guess of the P content was taken from the position of the diffusely scattered intensity from the InAs_1–*x*_P_*x*_ segment. The variation of the phosphorous content strongly influences the *Q*_*z*_ position of the InAs_1–*x*_P_*x*_ signal. An agreement between simulation and measurement was found within a variation of ±0.5% of the phosphorous content from the optimum value of 20%.

**Figure 4:**
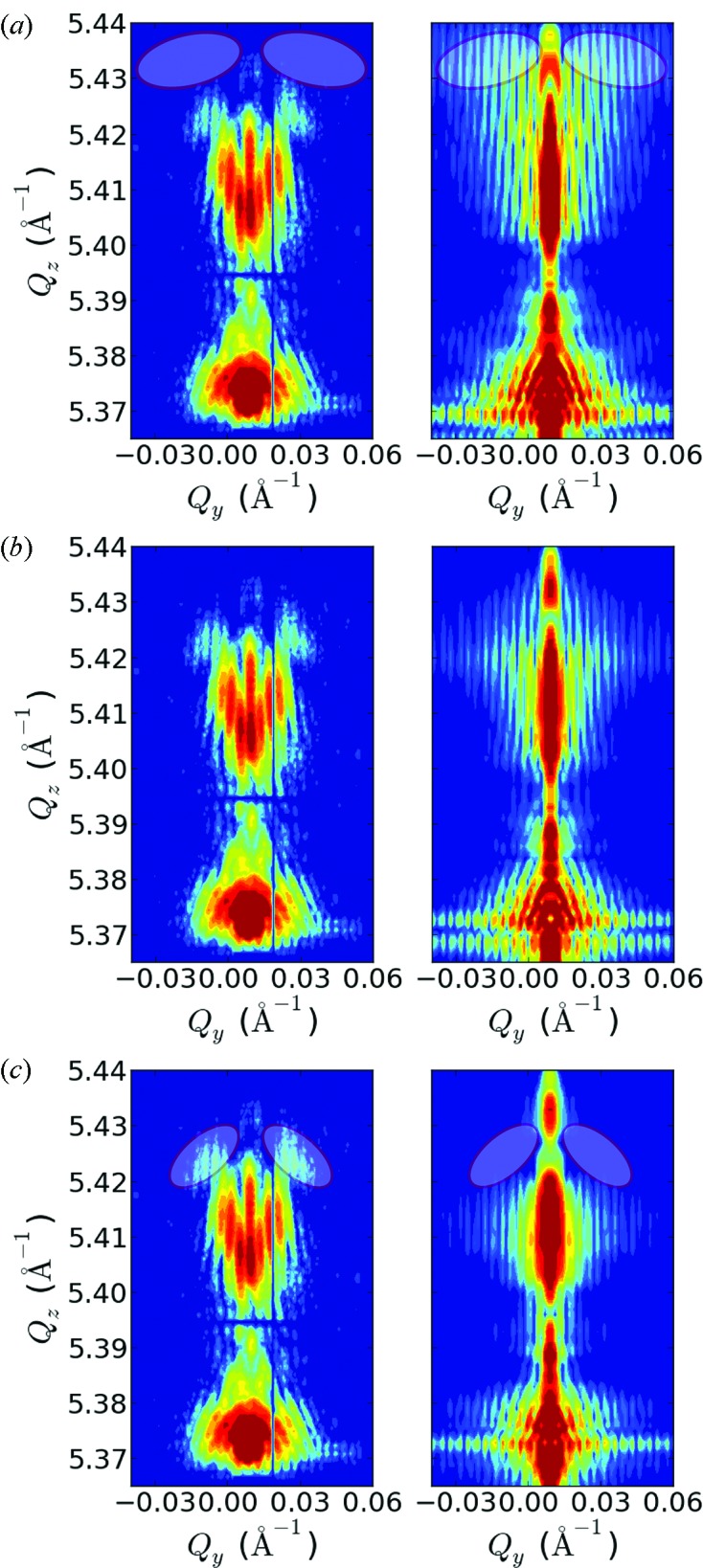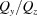 slices of the recorded three-dimensional RSM around the (333) InAs/InAs_1–*x*_P_*x*_ Bragg reflection from a single InAs_1–*x*_P_*x*_ segment in an InAs NW are shown on the left-hand side along with simulation results with different shell arrangements on the right-hand side. For comparison, the dynamic range of the intensities plotted as contour colours is always equal for both plots. Moreover, the dynamic range was chosen according to the analyzable intensity range of the measurement. Simulation parameters are equal for each simulation: NW diameter 175 nm, P content 20%, segment thickness 43 nm. In (*a*) a simulation without shells is shown, whereas in (*b*) shell thicknesses for the inner and the outer shell of 1 nm and 2.5 nm were used. In (*c*) both shells were chosen to be 4 nm thick.

**Figure 5:**
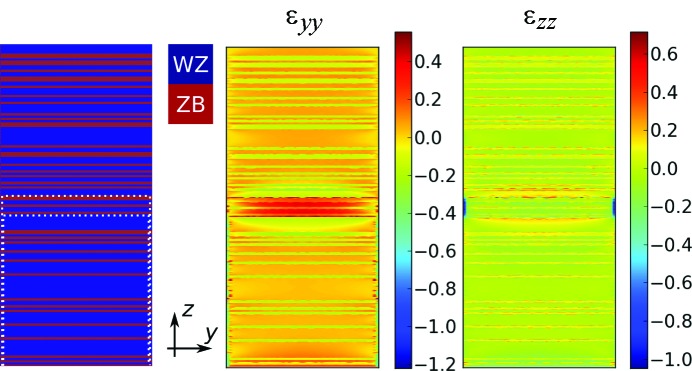
FEM simulation results for a P content of 20%, a segment thickness of 43 nm and a shell thickness for the inner and the outer shell of 1 nm and 2.5 nm, respectively. ∊_*yy*_ is the strain in the lateral direction and ∊_*zz*_ is that in the growth direction, given in %. The outer diameter of the simulated NW is 175 nm and the NW’s length is 700 nm. Note that the axes are not on the same scale. Moreover, the WZ/ZB distribution function used in this simulation is illustrated on the left-hand side.

The remaining NW dimensions can be quantified with less precision. The InAs_1–*x*_P_*x*_ segment length, for example, can already be varied by ±4 nm around the optimal value of 43 nm, and still an acceptable agreement between simulation and measurement is found. Similarly, the thicknesses of the inner InAs_1–*x*_P_*x*_ and the outer InAs shells have a more subtle influence on the simulations, as is illustrated in Fig. 4 ▶. Although the shells are very thin, neglecting them does significantly change the strain distribution, and therefore it is important to take them into account. Fig. 4(*a*) ▶ shows a simulation performed without shells, where the agreement of simulation and measurement is poor at higher *Q*_*z*_ values: above 5.425 Å^−1^ the intensity values of the simulation are higher compared with the measurement. On the other hand, simulating thicker shells results as well in a poorer agreement between simulation and measurement. This is illustrated in Fig. 4(*c*) ▶, where the regions with very poor agreement are again indicated. The optimum inner- and outer-shell thicknesses were found to be 1 nm and 2.5 nm, respectively. The strain values from the FEM simulation with these parameters are illustrated in Fig. 5 ▶, and the resulting simulated intensity pattern compared with the measurement is shown in Fig. 4(*b*) ▶.

In summary, the phosphorous content and the outer NW diameter influence the simulated signal stronger than the thickness of the InAs_1–*x*_P_*x*_ segment and the shell thicknesses. The results show a large axial/radial growth ratio producing only very thin shells.

**Figure 6:**
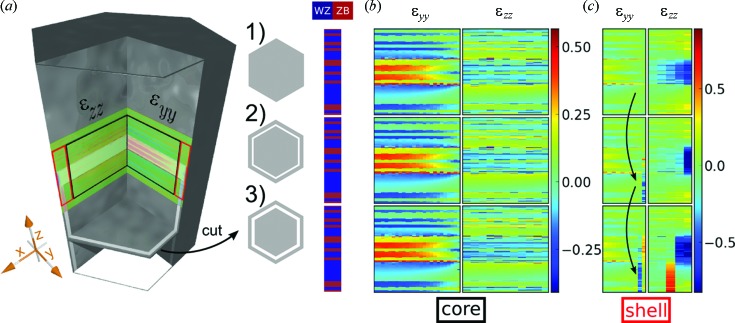
The strain in and around the InAs_1–*x*_P_*x*_ region is shown for simulations with different shell arrangements, indicated in cuts through the wire, shown in (*a*), for a NW without shells (1) and for a NW with two different shell dimensions (2, 3); in (2) an inner-shell thickness of 1 nm and outer-shell thickness of 2.5 nm and in (3) an inner-shell thickness of 4 nm and outer-shell thickness of 4 nm. For a better visualization the core region and the shell region of the NW are plotted separately, as depicted in (*a*) with black and red boxes. (*b*) ∊_*yy*_ and ∊_*zz*_ in the core region, *i.e.* in the black boxes. (*c*) ∊_*yy*_ and ∊_*zz*_ in the shell region, *i.e.* in the red boxes. The given strain values are always relative to the according relaxed ZB and WZ, InAs and InAs_1–*x*_P_*x*_ lattice parameters.

To illustrate the influence of different shell parameters and also the WZ/ZB segment distribution on the strain variation inside the NW, Fig. 6 ▶ shows a comparison of different FEM simulations with different shell dimensions. This results in a change of the strain values close to the circumference of the NW. One can clearly see that not only is the InAs_1–*x*_P_*x*_ segment strained but also the region around it as well as the thin shells. The highest strain values perpendicular to the NW growth direction (of the order of 0.6%) are located in the NW core region of the hetero-segment, but also above and below the segment in the InAs region. While the hetero-segment exhibits tensile strain, the regions above and below are compressively strained, see Fig.6(*b*) ▶. Owing to relaxation at the side walls, the strain values close to the NW border decrease compared with the NW centre. The shells influence the strain distribution particularly in these regions, and strains are underestimated in the simulation neglecting the shells. Already the introduction of a thin core-shell structure increases the strain values in the region near the NW side walls, leaving the strain in the core region virtually unaltered. An increase of the shell thicknesses is enhancing this effect, see Fig. 6(*c*) ▶. Thus, by changing the structural parameters one can achieve a tailored strain distribution. Moreover, the different WZ/ZB lattice constants locally introduce strain changes of the order of 0.2 to 0.3%, as can be seen in Fig. 6 ▶. This cannot be neglected compared with the strain values due to the hetero-segment. Simulations of the scattered intensity distribution from FEM results with different random distributions of WZ and ZB segments give very similar results, therefore we are not able to determine the particular segment distribution in the illuminated NW segment. However, not taking into account the strain caused by the polytypism results in an incorrect overall peak position. For a tight control of the strain distribution, it will thus be extremely beneficial to control the WZ/ZB polytypism during growth (Bolinsson *et al.*, 2011 ▶; Spirkoska *et al.*, 2009 ▶).

**Figure 7:**
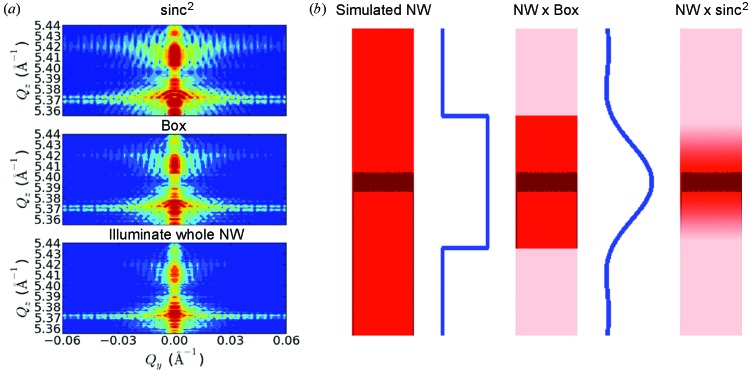
(*a*) Influence of the illumination function on the simulated intensity distribution, illustrated with three different simulations. To allow for a proper comparison, the dynamic range of the plots is set equal. (*b*) Graph of the illumination functions along with the shape function of the simulated NW (InAs in red and InAs_1–*x*_P_*x*_ in dark red). The plots are scaled in the *z* direction. Moreover, the effect of multiplying the illumination functions to the scattering strength of the simulated NW is illustrated.

To account for the finite size of the focused beam a simple approach was used, where the Fourier coefficients of the crystal polarizability in the simulated NW’s growth direction were multiplied by an illumination function with minimum and maximum values of 0 and 1, respectively. This approach is reflecting the real physical situation of the nano-focused scattering experiment, where the focused beam and its transverse coherence length are at the same order of magnitude due to a partial illumination of the Fresnel zone plate during the experiment. Since we did not determine the exact shape and phase of the focused beam in this study, three simple illumination functions were compared: a box function, a sinc^2^ function and a constant illumination of the whole simulated NW as depicted in Fig. 7(*b*) ▶. Fig. 7(*a*) ▶ illustrates a comparison of the resulting RSMs from simulations with different illumination functions. The results for the sinc^2^ and Box functions are rather similar, *i.e.* we are not sensitive to details in the illumination function. However, by neglecting the finite beam size and calculating the scattered intensity distribution from the whole simulated NW (corresponding to a plane incident wave illuminating the whole simulated NW), very poor agreement between measurement and simulation was achieved. This can be seen around 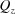 = 5.375 Å^−1^ where the features from the measurement have not been reproduced, *e.g.* the diagonal intensity stripes in the measurement are not reflected in the simulation. A more precise approach for the estimation of the focused beam’s properties was used by Mastropietro *et al.* (2013 ▶), where the beam size and shape was calculated from the estimated undulator source size and the utilized FZP, and Schropp *et al.* (2010 ▶) have analyzed the beam properties using ptychography experiments, resulting in a detailed picture of the focused beam’s shape and coherence properties. Taking into account those beam profiles does not, however, improve our simulation results any more.

## 6. Conclusion   

By using X-ray diffraction with a nano-focused beam, we have performed a detailed analysis of the strain distribution in and around a single axial InAs_1–*x*_P_*x*_ hetero-segment in an InAs NW. Quantitative results were deduced using simulations of the scattered intensity distribution, resulting in a complete picture of the investigated structure. The hetero-segment’s chemical composition, the geometrical parameters and also the thicknesses of the thin radial shells were deduced for a single as-grown nanowire. Additional ensemble-averaged experiments were used to evaluate the WZ/ZB ratio of the fabricated nanowires as well as the average crystallite’s segment length. The strain distribution was found to be rather inhomogeneous due to the combination of axial and radial growth in the self-seeded particle-assisted growth mode. The precise measurement of the strain distribution in NW heterostructures provides the knowledge for the fabrication of NW devices with dedicated physical properties.

## Footnotes

1 We use Miller and Miller–Bravais reciprocal lattice point index notation to distinguish cubic and hexagonal crystal polytypes, respectively.

2 In the construction of the FEM mesh, the maximum length of the longest mesh elements edge was set to 3 nm in the shell region, whereas in the rest of the simulated structure it was chosen coarser at 6 nm. To reduce computational cost the model contained only one-quarter of the NW; the result of the full NW was then reconstructed using symmetry operations according to the wire geometry and crystal structure.
